# Poly(Vinyl Alcohol)-Based Nanofibrous Electrospun Scaffolds for Tissue Engineering Applications

**DOI:** 10.3390/polym12010007

**Published:** 2019-12-18

**Authors:** Marta A. Teixeira, M. Teresa P. Amorim, Helena P. Felgueiras

**Affiliations:** Centre for Textile Science and Technology (2C2T), Department of Textile Engineering, University of Minho, Campus of Azurém, 4800-058 Guimarães, Portugal; martaalbertinateixeira@gmail.com (M.A.T.); mtamorim@det.uminho.pt (M.T.P.A.)

**Keywords:** poly(vinyl alcohol), electrospun scaffolds, tissue engineering, biocompatibility, mechanical stability

## Abstract

Tissue engineering (TE) holds an enormous potential to develop functional scaffolds resembling the structural organization of native tissues, to improve or replace biological functions and prevent organ transplantation. Amongst the many scaffolding techniques, electrospinning has gained widespread interest because of its outstanding features that enable the production of non-woven fibrous structures with a dimensional organization similar to the extracellular matrix. Various polymers can be electrospun in the form of three-dimensional scaffolds. However, very few are successfully processed using environmentally friendly solvents; poly(vinyl alcohol) (PVA) is one of those. PVA has been investigated for TE scaffolding production due to its excellent biocompatibility, biodegradability, chemo-thermal stability, mechanical performance and, most importantly, because of its ability to be dissolved in aqueous solutions. Here, a complete overview of the applications and recent advances in PVA-based electrospun nanofibrous scaffolds fabrication is provided. The most important achievements in bone, cartilage, skin, vascular, neural and corneal biomedicine, using PVA as a base substrate, are highlighted. Additionally, general concepts concerning the electrospinning technique, the stability of PVA when processed, and crosslinking alternatives to glutaraldehyde are as well reviewed.

## 1. Introduction

Tissue engineering (TE) provides a new way to recover lost physiological functions. It comprises the construction of natural and/or synthetic structures, allows the combination of these materials with growth factors and/or signaling molecules to modulate cell proliferation and differentiation, and develop constructs resembling the extracellular matrix (ECM) [1]. These features overcome the limitations of auto- and allografts, by reducing post-surgical recovery periods and preventing the use of expensive therapies [2,3].

Fibrous scaffolds with specific characteristics as aligned ultrafine fibers capable of encapsulating functional dopants, light-emitting dyes, drugs and biomolecules, have been produced using electrospinning [4,5]. For instance, uniaxially aligned nanofibers have shown higher efficiency to guide cell migration than randomly organized fibers; however, they have not been successful in all applications, in specific wound treatments their irregular shape is known to compromise the healing process [6]. This technique allows to control the morphology, porosity and fiber diameter of the scaffold, to fit the requirements of specific applications, by adjusting the processing parameters, using simple and low maintenance settings [7]. These important adaptable characteristics make the electrospinning technique particularly suitable for the production of biomimetic nanofibrous scaffolds, providing new generation strategies to restore, maintain or improve tissue functions [8,9]. These essential trademarks instigate cell adhesion, attachment, proliferation, differentiation and migration [10], while meeting the material requirements of biocompatibility, biodegradability, mechanical stability and nutrient transport throughout the assembly [11,12,13,14,15]. 

Over the years, various biodegradable polymeric scaffolds have been used for soft and hard tissues repair/substitution. In fact, their application in biomedicine has progressively increased, with proven value in reconstruction of multi-tissue organs, tissue interfaces and even structural tissues, including bone, cartilage, tendons, ligaments and muscles [10,11]. They have also been associated with fibrous constructs for nerve, heart and vascular systems, both individually or blended with other biopolymers [16,17,18]. Between the many polymer-based scaffolds already studied, poly(vinyl alcohol) (PVA) scaffolds are known to provide mechanical stability (high tensile strength and elongation at break), flexibility and slow degradation kinetics compared to scaffolds made of natural polymers [19,20]. These properties have conferred PVA-based scaffolds with the capacity to absorb more strain during muscles/bone mechanical loading and to support more easily the strain changes caused by cardiac contractions [21]. In neural tissues, the scaffold microstructure, its three-dimensionality and aligned fibers, is as important as its biological properties. Even though many materials and techniques have been employed to this purpose, PVA-based electrospun nanofibers have been shown to meet all the requirements as they can be tuned to fit specific alignments, porosity and architectures, while maintaining their flexibility and biological features [22]. The clear and transparent nature of PVA has also attracted attention to produce bioengineered corneal equivalents. A healthy cornea contributes to two-thirds of the total refractive power of the eye; as such, a corneal equivalent should be able to transmit most of the visible light without compromising its mechanical integrity [23]. This polymer’s ability to be processed with various degrees of hydrolysis, a property intimately related with its degradation rate, has also raised PVA’s profile when it comes to drug delivery systems.

In the present review, the advantages of using polymeric matrices based on PVA for the construction of electrospun scaffolds for the most varied TE purposes will be enumerated. The impact of the electrospinning technique and crosslinking steps for the successful construction of such scaffolds will also be highlighted. Relevance will be given to recent advances in TE in which applications of PVA-based electrospun scaffolds have proven promising in providing health benefits. The goal of this review is to establish the importance of PVA in biomedicine. Even though reviews have been published about electrospinning, to this moment none has focused on the TE breakthroughs introduced by the processing of PVA in the form of nanofibers using this technique. 

## 2. Electrospinning in the Production of Nanofibrous Scaffolds

Electrospinning was developed by Formhals in 1934 but the interest in this technique only grew in the 90s with the advances in nanotechnology [24]. This technique’s principle is simple, in which a polymer solution, natural or synthetic, is pumped through a high voltage source at constant rate [25]. It is a direct extension of the electro-spraying phenomenon, which is based on physical and electrical mechanisms [26]. Electrospinning is a versatile method to yield non-woven fibrous structures with fiber diameters ranging between few nanometers to lower than one micrometer, a size that is otherwise difficult to attain using conventional spinning techniques (i.e., melt spinning, wet spinning, dry spinning, etc.). Besides, it produces fibers with long lengths, high surface area per unit volume [12,13,27,28,29], superior mechanical properties and it allows fiber functionalization for a variety of purposes [14]. Compared to other techniques used for nanofibrous fabrication such as phase separation, self-assembly, template synthesis, mechanical drawing, melt blowing, hydrothermal processing, centrifugal force spinning and bicomponent extrusion, this method is most effective in producing nanofibers with an uniform structure [30].

The control of determined electrospinning processing parameters influences directly the fibers’ diameter, surface morphology and texture of the fibers. These parameters include, the intrinsic properties of the polymer solution (e.g., concentration, electrical conductivity, surface tension and viscosity), the operational settings (e.g., the strength of the electrical field, distance between the spinneret and collector and rotating speed of the collector and feeding rate of the polymer solution) and the environmental conditions (e.g., humidity and temperature) [31,32,33]. 

The polymer concentration in the solution determines its feasibility of processing into nanofibers and has an important effect in the final morphology. An increase in the solution concentration leads to an increase in the fiber diameter and uniformity [34]. However, increasing the concentration beyond a critical value hampers the flow of the solution through the needle tip (the polymer solution dries and blocking the needle-tip), which ultimately results in defective or beaded nanofibers. Another fundamental parameter for nanofiber formation is the conductivity. Electrospinning requires the transfer of electric charges from the electrode to the spinning droplet. Therefore, solutions with low conductivity will not be able to form a Taylor cone due to the absence of charge [35]. Increasing the conductivity of the solution will subject the fiber jet to a greater tensile force, which yields non-beaded fibers with reduced diameter [36]. The electric conductivity is affected by polymer and solvent type, polymer concentration and temperature [34]. The solution feeding rate influences the jet velocity and transfer rate. For optimal solvent evaporation and, consequently, to obtain solid/uniform nanofibers, lower feeding rates are desirable. High feeding rates may generate beaded, large diameter fibers since there is insufficient time for solvent evaporation (before reaching the collector) [3]. Hence, the distance between the tip and the collector is another factor controlling the fibers diameter and morphology [37].

The electrospinning process has been used as an efficient and affordable processing technique to fabricate nanofibers. The most common materials applied in the production of electrospun fibers are natural or synthetic polymers and hybrid polymeric blends (Table 1) [38]. Synthetic polymers have great flexibility during synthesis, allow chemical modifications and possess predictable and reproducible mechanical and physical properties, such as tensile strength, elastic modulus and a degradation rate that mimics the native tissues [39,40]. These polymers are often cheaper than natural components, allowing mass production of tailored scaffolds; however, due to their low hydrophilicity and absence of cell recognition sites synthetic polymers lack cell affinity [39]. Compared to synthetic polymers, natural polymers exhibit better biocompatibility and lower immunogenicity, and some even display intrinsic antibacterial properties [41,42,43]. Yet, their physical and mechanical features are more difficult to modify. 

For TE applications, polymeric nanofibers are the most suitable platforms due to their exceptional physical, chemical and mechanical properties, making them desirable for cell–cell and cell–matrix interactions [3]. These materials allow nanofibrous scaffolds to be produced with high porosity, high pore interconnectivity, thin fiber diameter and a controllable and uniform structure, adjusted to the requirements of the injured site [30].

In the medical and pharmaceutical fields the need for novel nanofiber designs that include hollow, porous, multichannel tubular, shape of necklace, core-sheath, nanowebs, nanowire, multilayer structures, among others, has increased considerably over the years since these new architectures respond better to certain applications [38,44,45,46]. By combining new electrostatic and magnetic strategies to the electrospinning technique, advances have been made towards the production of aligned, spiral, tubular and sheath membranes that respond to specific demands in TE [8]. Aligned fibers exhibit greater strength in one direction, being more useful for tendon and ligament regeneration. On its turn, random nanofibers can be adjusted for an improved stiffness and resistance in all directions, becoming more useful for skin and cartilage applications [47].

**Table 1:** Typical morphology and assembly of natural, synthetic and blended electrospun nanofibers (adapted with permission from [48]).

**Composition**	**Natural (Nt)**	**Synthetic (St)**	**Blended**
	Collagen	Poly(vinyl alcohol)	Nt + St
	Chitosan	Poly(ε-caprolactone)	Nt + St + Coating
	Silk	Poly(lactic acid)	
	Alginate	Poly(lactic-co-glycolic acid)	
**Morphology**	**Solid** 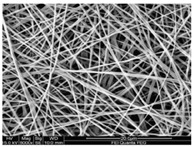	**Porous** 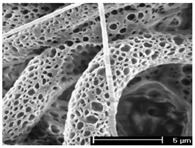	**Core-Shell** 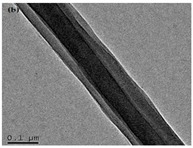
**Assembly**	**Random** 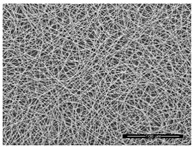	**Aligned** 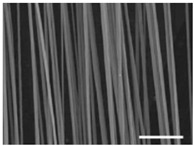	**Layered** 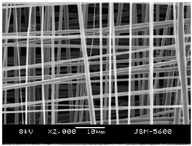
	**Yarn** 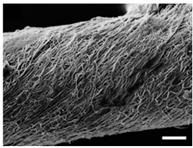	**Hollow** 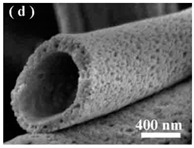	**Fiber Bundle** 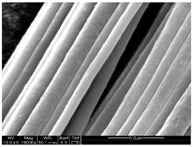

**Figure 1:**
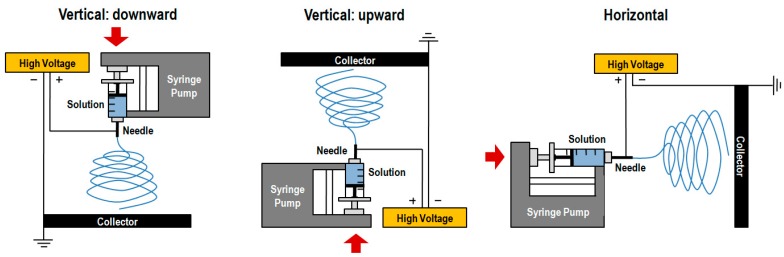
Representation of vertical downward, vertical upward and horizontal electrospinning setups.

**Figure 2:**
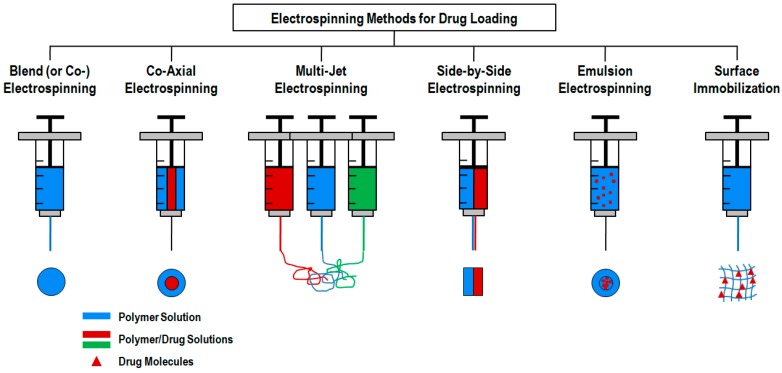
Schematics of the electrospinning methods available for drug and biomolecules loading into nanofibers (adapted with permission from [51]).

The simplest electrospinning apparatus requires a single-needle spinneret to draw the solution out and form fibers. Typically, there are two types of electrospinning setups: horizontal and vertical (upward and downward) as depicted in Figure 1 [46]. However, this process is very time consuming, limiting the potential large-scale application of the resulting products [44]. The production rate of a conventional electrospinning system is less than 10 g h^−1^ of nanofibers, depending on polymer concentration and operating conditions [49]. Thus, various researches have been devoted to the design of innovative and versatile electrospinning configurations capable of fabricating complex constructs more easily [50]. In fact, there are already several electrospinning systems available that include blend (or co-), side-by-side, multi-jet, co-axial and emulsion electrospinning (Figure 2) [41]. Each method displays unique features that allow, for instance, the production of scaffolds with high drug loading capabilities, increased initial burst, sustained release, prolonged circulation, etc. [36].

The blend (or co-) electrospinning method is the most simple and cost-effective, as it only requires the mixing of polymer solutions and the traditional electrospinning setup [41]. In specific biomedical cases, this technique incorporates drugs/biomolecules by blending them with the polymer solution [36]. It offers high drug loading, homogenous spreading of drug molecules and an enhanced initial burst. Furthermore, this method allows the use of special spinnerets capable of generating efficient drug delivery structures as core-sheath, hollow fibers, porous fibers and multichannel microtubes [52]. However, during this process, the bioactive agents may lose their bioactivity in the presence of the solvents or when exposed to the electric field [53]. Another major drawback of this method is the agglomeration of the bioactive agents on the surface of the fibers, triggering an initial burst release that may increase the probability of site toxicity and, thus, reducing the effective lifetime of the medical device [54].

The co-axial electrospinning method is very similar to the traditional electrospinning process with the exception of using two concentrically aligned capillaries, instead of one, connected to a high voltage source to enforce the formation of fibers with a core-shell structure [45,55]. Co-axial electrospinning is a popular route to obtain hollow nanofibers. It is very important for drug controlled release since this methodology is capable of circumventing the limitations of single-nozzle electrospinning by encapsulating fragile, water-soluble bioactive agents or co-encapsulating multiple drugs with different solubilities in an one-step process [3]. The shell polymer not only contributes to the sustained and prolonged release of the therapeutic agent but also protects the core ingredient from direct exposure to the biological environment. The main advantage of this method over conventional electrospinning is that the fibers are fabricated from two separate solutions, minimizing the interaction between aqueous-based biological molecules and the organic solvents in which the polymers are mainly dissolved [54]. Since this method requires the precise control of process variables such as interfacial tension and viscoelasticity of the two polymers, the design/process complexity increases considerably [53].

A simple approach to enhance the production rate of nanofibers was developed by increasing the number of nozzles used, which includes not only needles but also tips, holes and channels. This approach is known as multiple-jet electrospinning and can be classified into (i) a single nozzle with multiple jets, (ii) multiple nozzles with a single jet exiting from each nozzle or (iii) multiple nozzles with multiple jets exiting from each nozzle [44]. The multiple-jet electrospinning overcomes the drawbacks of single jet spinning in terms of productivity and offers the potential for mass production by means of a simple and versatile setup, with greater control of fiber distribution [41]. Still, the use of multiple jets raises other issues including jet repulsion, the non-uniformity of electrical fields on the nozzle tips, lower process controllability and deterioration of fiber quality [56]. To address these problems, another configuration of this method has been engineered and classified as free surface electrospinning (also referred to as tipless, needleless or unconfined electrospinning) [57]. In free-surface electrospinning, the solution flows unconfined over a free surface, in which the formation and self-organization of the multiple jets occurs. Here, the nanofiber’s production rate is 12 times superior to the conventional electrospinning equipment; however, the fiber’s diameter distribution is not uniform [44,46]. This configuration eliminates the problems of solution clogging and jet interference, and has a much higher yield compared to a similar sized multiple nozzle setup [58]. Still, technological problems have been identified as barriers to the widespread use of this methodology, including the need for high voltage power supplies and rapid solvent evaporation, as well as high costs [59]. This method is very important to most cancer treatments that involve the exploitation of multiple drugs (i.e., solid tumors) or demand a combination of therapies. Through electrospinning, it is possible to load different biomolecules into a single system by using either a multi-jet or multi-layer approach [36,60]. Despite the emergence of more recent free surface techniques, research in nozzle electrospinning is still ongoing, owing it to the simplicity of the setup and better fiber control [44].

Another very interesting method to electrospun two polymers or a polymer and a drug/biomolecule of interest that are not soluble in a common solvent, is the side-by-side approach [36]. In this method, the carrier/polymer solution and the biomolecules are loaded in a separate spinneret. While applying the electrical field, fibers with a distinct upper and lower layer are deposited on a common target. This approach can be used to delay the initial burst of the drug molecules [61]. It allows maximum exposure of both components on the surface, offering a high surface area, while allowing an increased adsorption of various reactants and products and generating a controlled morphology [62].

A great number of limitations from the previous methods can be overcome using emulsion electrospinning [50]. This method allows for two immiscible solutions to be electrospun into a single fibrous scaffold and, therefore, maximize the potential for TE and drug delivery applications [63,64]. This is an effective strategy to produce cost-effective nanofibers (mass production) [65]. Unlike the traditional electrospinning process that requires highly viscous solutions, the emulsion electrospinning can produce optimal nanofibers using dilute polymer solutions and polymers of low molecular weight [63]. Despite the emulsion electrospinning method requiring the same basic set up as conventional electrospinning [30,63], this technique has been applied to engineer core-shell structured nanofibers [66]. The drug and polymer are dissolved in the appropriated solvents, eliminating the need for a common solvent [54]. Encapsulating bioactive agents in the core using water/oil phase or oil/water phase (W/O or O/W) emulsions prevents the side-effects associated with organic solvents contact, one of the major contributors to bioactive agents denaturation [66]. Hence, through encapsulation, the biological agent is protected in the core fiber and its release rate can be managed by controlling the structure and composition of the shell [66,67].

## 3. **Poly(Vinyl Alcohol**)

**Figure 3:**
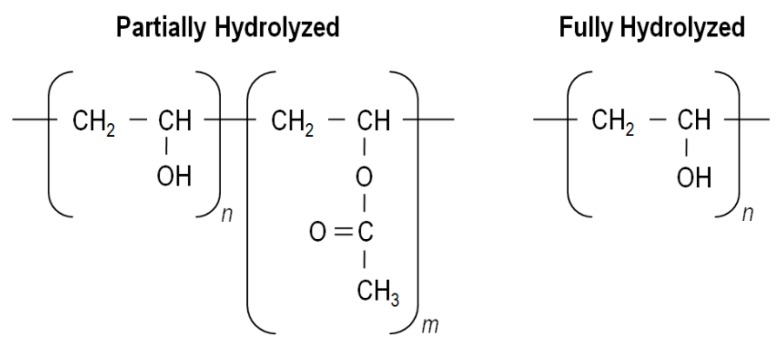
PVA partially hydrolyzed and fully hydrolyzed chemical formula (adapted with permission from [78,79]).

PVA was the first synthetic colloid to be prepared by Hermann and Haehnel in 1924 [68]. Its chemical structure is relatively simple, characterized by a main chain constituted by C–C bonds with hydroxyls and acetate groups on its laterals. The number of these groups is related with its synthesis process. The first step consists in the polymerization of the vinyl acetate monomer into poly(vinyl acetate) (PVAc) followed by hydrolysis of the acetate groups of the PVAc. Since this conversion may not be complete, different degrees of hydrolysis may be attained. PVA is characterized as partially hydrolyzed when it presents about 80.0%–98.5% of –OH groups, as highly hydrolyzed in the presence of >98.5% of –OHs, and in the absence of any acetate group is denominated as totally hydrolyzed (Figure 3). Depending on the catalyst used, PVA can be hydrolyzed via alkaline hydrolysis, aminolysis and acidolysis [69,70,71,72,73]. At an industrial scale, this process is usually done by alkaline hydrolysis through ester substitution with methanol in the presence of sodium hydroxide (NaOH) [74]. The degree of hydrolysis, the molecular weight (influenced by degree of polymerization) and tacticity affect the chemical and physical properties of the polymer among which solubility, crystallinity, biodegradability, etc. [19,33,74,75,76].

PVA with low degree of hydrolysis shows higher solubility in water at low temperatures compared to PVA with high degree of hydrolysis. Indeed, residual acetate groups (hydrophobic in nature) can weaken the intra- and intermolecular hydrogen bonds of adjoining –OH groups, making it necessary to raise the temperature well above 70 °C to completely dissolve high degree of hydrolysis PVA in water systems [19,77]. This phenomenon, makes PVA particularly desirable as a hydrophilic additive in blend scaffolds, since PVA does not easily leach (low solubility) into water or culture media at room or body temperature (37 °C) [77].

Various studies have shown the influence of the molecular weight in the properties of the PVA polymer. According to Hajij et al. high molecular weight PVA produced blend films with greater tensile strength and elongation at break, and that high degree of hydrolysis confers great rigidity to the blended films [80]. Lee et al., also concluded that high molecular weight PVA displays a highly crystalline structure, improved thermal stability and superior mechanical properties than low molecular weight PVA [72]. The polymer crystallinity is also influenced by the degree of hydrolysis. Since acetate groups are larger than –OH groups, PVA with a low degree of hydrolysis exhibits lower crystallinity. As such, a highly hydrolyzed PVA is more crystalline than partially hydrolyzed PVA and presents higher glass transition and melting temperatures [81,82,83,84,85,86]. The effect of the molecular weight and the degree of hydrolysis on the properties of PVA is highlighted in Table 2.

Even though PVA is one of the oldest synthetic polymers, it continues to be frequently used in advanced biomedical applications because of its excellent biocompatibility, biodegradability, excellent transparency, film forming abilities, thermo-stability and chemical resistance [81,82,83,84,85,86]. Nevertheless, its restricted strength, low thermal stability and instant dissolution or excessive swelling in aqueous environments has limited its application and made the crosslinking process indispensable [88,89,90]. Crosslinking implies the formation of chemical bonds between different molecular chains to generate strong 3D networks [91]. In PVA, it can be accomplished via chemical reaction (e.g., free-radical polymerization, chemical reaction of complementary groups, high energy irradiation or enzymatic reaction) or by physical reaction (e.g., ionic interaction, crystallization of the polymeric chain, hydrogen bond, protein interaction or design of graft copolymers) [81]. The most frequent chemical agents or crosslinkers applied to PVA are the dialdehydes, diisocyanates, dicarboxylic, tricarboxylic and boric acids (Table 3) [92]. Even though there are many options, glutaraldehyde (GA) is by far the most common crosslinker used in PVA processing. GA has been considered the most effective in stabilizing various biomaterials, is easily accessed, and is low cost [93]. In recent decades, interest in physical crosslinking (e.g., freezing-thawing, elevated temperatures, ultraviolet (UV) radiation) has increased considerably since, contrary to the traditional chemical crosslinking agents, physical methods are non-toxic and do not affect the nature of biomolecules like proteins, drugs and cells [81,91]. Lately, it has even been attempted to initiate the crosslinking process whist the electrospun fibers are being generated, resorting to both physical and chemical crosslinkers (UV-light, maleic anhydride and direct incorporation of GA) [94].

## 4. Tissue Engineering Applications of PVA-Based Nanofibrous Scaffolds

**Figure 4:**
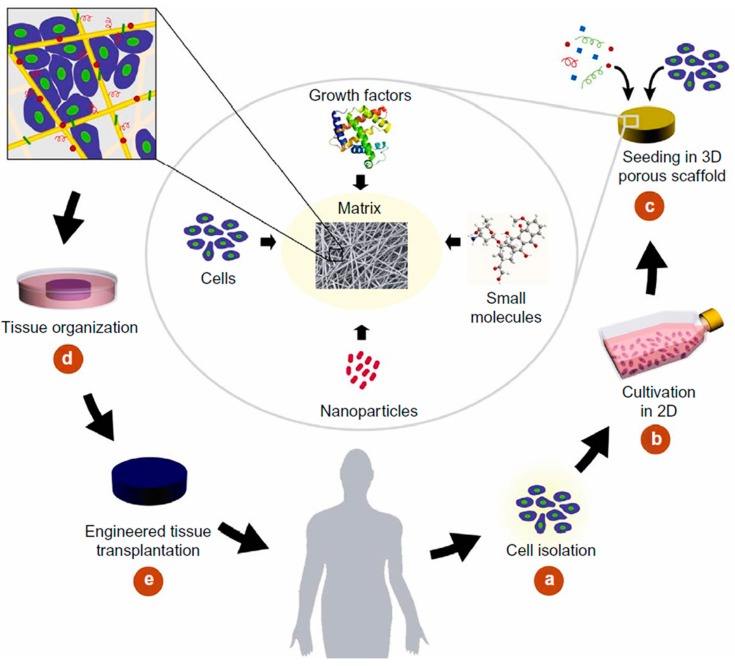
Seeding of cells into fibrous electrospun scaffolds for tissue engineering (TE) applications (adapted with permission from [96]).

The growing need for tissue substitutes has motivated an intense research in tissue reconstruction and regeneration [95]. For tissue defect treatment, autografts have been appointed as a “gold-standard”. However, limited supply and donor site morbidity remain major drawbacks of their use. Allografts and xenografts are high risk factor treatments because of the immune response and disease transmission associated complications. For these reasons, TE scaffolds have gained more and more attention with time, and are now considered an important option for next-generation tissue therapies [96]. Rather than simply introducing cells into a diseased/affected area to restore its functions, in TE cells are often seeded into a temporary platform or scaffold that promotes the reorganization of the tissue (Figure 4). For successful tissue regeneration, an ideal TE scaffold should be biologically functional and mechanically stable. The scaffold biocompatibility, porosity and biodegradability, which include the absence of toxicity presented by its degradation by-products, are the most important factors, which condition the production of scaffolds. These properties are dependent on the selection of the material, the scaffold architectural design and its ability to establish and maintain cell–material interactions [12,95,97]. These remarkable features can be achieved with the electrospinning technique. This technique allows the conversion of a broad range of materials into fibers and to explore different morphologies and structures, so that scaffolds with peculiar properties may substitute the natural ECM.

The architectural design of the TE scaffolds is very important for their success. The growing preference towards 3D porous structures over the traditional two dimensional (2D) materials has allowed exceptional features and large surface area-to-volume ratio scaffolds to be engineered, with light weight, flexibility, high porosity, spatial alignment or random deposition (depending on its purpose), and an interconnected network [88,98]. Light, insoluble and stable PVA scaffolds can be produced with a 3D rendering and modified to promote the adhesion of healthy human mesenchymal stem cells (hMSCs) within the 3D micro-niches of the construct. hMSCs are capable of differentiating into osteocytes, adipocytes, chondrocytes and other cells under specific conditions (multipotent cells). They can be isolated from various tissues, including embryonic stem cells (ESCs) and induced pluripotent stem cells (iPSCs), and implanted into debrided defected sites or used in matrices like scaffolds, to prevent chondrocytes from losing their cartilage-like properties and acquiring a de-differentiation status during in vitro expansion [99]. Unlike 2D substrates, the 3D scaffolds are considered more biocompatible, enabling optimal cell penetration, adhesion and viability in vivo, and thereby revealing a promising potential in devising stem cell-based tissue regenerative therapies [88]. Many studies have demonstrated that conventional 2D biomaterials (e.g., fibrous sheets and biofilms) cannot maintain the cell phenotypes derived from complex multi-cellular tissues due to the dramatic physical and chemical differences between 2D in vitro culture and the native cellular microenvironment [98].

Apart from the scaffold’s architecture, the surface properties are also crucial for a successful implantation and regeneration. Different surface modification approaches, including plasma treatment, wet chemical modification and surface graft polymerization, have been applied in electrospun scaffolds [96]. Plasma treatment introduces new chemical groups at the scaffolds’ surface, while promoting the alteration of its topography [100]. Partial hydrolysis by means of wet chemical modification (acidic or basic treatments), modifies the scaffolds’ surface by chemical scission of the ester linkages (a random process) and offers the flexibility necessary to modify the surfaces of thick nanofibrous meshes [101]. Surface graft polymerization is mostly based on the generation of surface free radicals, using UV radiation or plasma treatment to induce polymerization [96].

PVA-based nanofibrous scaffolds have shown a great potential in numerous TE applications, including bone, cartilage, skin, vascular, neural, corneal and as vehicles for the controlled delivery of drugs, proteins, growth factors, nanoparticles and even DNA [39,102,103]. In the following sections, the potentialities of PVA-based scaffolds, produced via electrospinning technique, are explored in some of the most pressing TE areas.

### 4.1. Bone

Bone injuries and defects, that include orthopedic diseases, are often a result of trauma, tumors, osteoporosis and even infections [104]. Some of these problems are relatively simple to treat but, certain pathological fractures and complex breaks, arising from tumors, malformations and osteoporosis, are a serious challenge [2]. Current therapies are not completely effective since problems such as the use of inappropriate devices, long recovery periods and complications with infections and non-unions, disease transition and expensive immune supportive drugs, continue to persist. Various alternatives have been proposed to respond in an effective way to these complications. For instance, electrospun scaffolds have been combined with growth factors and cells to repair bone injuries and generate an efficient support that mimics the ECM [105].

**Figure 5:**
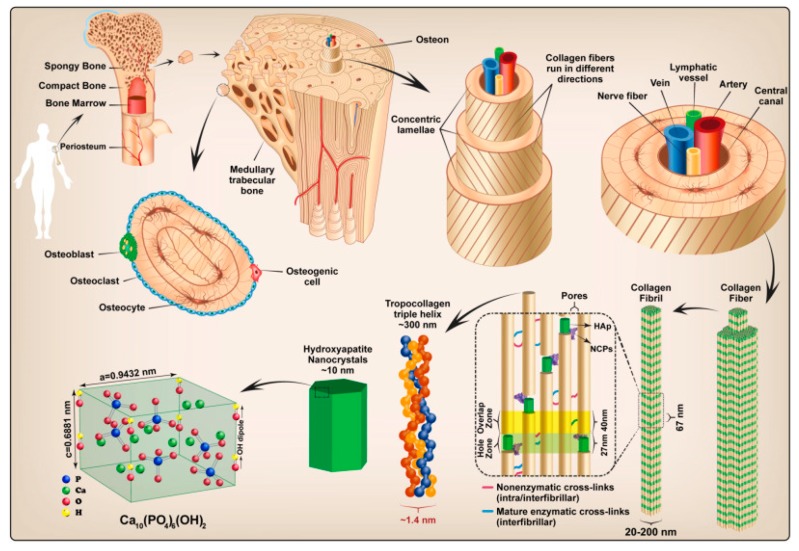
The highly specialized ultrastructure of bone tissue—organization and composition (adapted with permission from [110]).

Bone is comprised of four types of cells, namely osteoblasts, osteoclasts, osteocytes and bone lining cells, which dynamically regulate bone homeostasis [106], and is composed of collagen type II (Col II, organic phase) and hydroxyapatite (HA [Ca_10_(PO_4_)_6_(OH)_2_], mineral phase; Figure 5) [2,107]. HA has been widely explored and applied in composites for bone regeneration uses given it resembles the natural minerals found in bone, conferring osteoconductivity and structural integrity to the scaffold. Other calcium phosphate variants or bioglasses (BG) have also been used in bone therapies due to their biocompatibility [15,48]. These materials replace the inorganic part of natural bone, and their role is to accelerate the formation of bone-like apatite on the surface of the implant. However, HA has poor mechanical properties and cannot be used alone for bone repair and substitution. Instead, HA is used as reinforcement material to trigger tissue growth [108]. Composites made of ceramics and polymers result in materials endowed with the bioactivity of the calcium phosphate phase and the ductility of the polymers [109].

Development of novel scaffold materials that mimic functionally and architecturally the ECM is very important to meet the demands of the advances in bone TE. An ideal scaffold for bone repair/substitution should be (i) flexible, to adapt to any form and space and to be easily arranged into 3D constructs, (ii) its topography and morphology should promote cell adhesion, infiltration and growth and finally (iii) its degradation rate should be proportional to the rate of tissue regeneration, without inducing any toxic or inflammatory responses, in surrounding tissues or the whole host system, by its degradation byproducts [106].

There have been many studies describing the importance of polymers in the manufacture of scaffolds for bone repair and regeneration, resorting to the electrospinning technique. Shalumon et al. combined PVA and carboxymethyl chitin (CMC) in a nanofibrous scaffold and accessed the adhesion and proliferation of hMSCs. Data revealed the PVA/CMC scaffolds to support cell adhesion and proliferation, and to instigate mineralization by increasing the production of calcium phosphate on the surface [111]. Hydroxyethyl cellulose (HEC), a natural derived polymer, has also been combined with PVA. Chahal et al. designed a bead-free scaffold by optimizing the ratio between PVA and the cellulosic compound and showed that, aside from enhancing the spinnability of HEC, PVA also increased the mechanical strength of the nanofibers [112]. As the HEC percentage increased in the PVA/HEC scaffolds, the elastic modulus and tensile strength increased and the elongation at break decreased proportionally. They established that the PVA/HEC scaffolds favored human osteosarcoma cells spreading, attachment and proliferation [113]. By functionalizing the PVA/HEC scaffold with bone-like calcium phosphate (CaP), using 10 times fold concentrated simulated body fluid (SBF) solution, the tensile properties of the nanofibers were significantly enhanced, approximating the tensile strength and the elastic modulus of the trabecular and proximal femoral bones [114]. Similar observations were made with the addition of nanohydroxyapatite (nHA). The nHA mineralization enhanced the tensile strength and reduced the elongation at break of the scaffolds, while improving the overall wettability of the nanofibrous scaffolds and thus the osteosarcoma cells adhesion and proliferation [115]. Many studies have reported cellulose nanofibers (CNF) and nHA as excellent candidates for bone scaffolds production, considering CNF as the reinforcing nanofiller and nHA as the bioactive material [116,117,118]. Enayati et al. produced PVA, PVA/nHA and PVA/nHA/CNF based scaffolds and showed that the fiber diameter, the degradation rate and the elongation at break decreased after the nanofillers addition, even though the tensile strength increased [2]. Highly porous (64%) electrospun scaffolds made of PVA/polycaprolactone (PCL) blended with nHA have been produced with a swelling ratio of 141%. The introduction of the inorganic material disturbed the crystallinity of PVA, reducing the enthalpy of fusion. The biocompatible PVA/PCL/nHA scaffold enhanced the adhesion and proliferation of MG-63 osteoblast-like cells [108]. HA-based bioceramics (HAB) have been combined with PVA/PCL to address the difficulty to correct bone defects in complicated fractures or tumors in the craniofacial region. The scaffolds’ influence on bone marrow skeletal hMSCs and dental pulp stem cells (DPSC) was examined in vitro. Cell attachment was improved from day 1, with near 70% more cells seeded onto the composite scaffold compared to the individual polymers (PVA and PCL) [119]. Composites formed of PVA and polyvinylpyrrolidone (PVP) with different concentrations of bioactive nHA have been successfully fabricated with a 3D open-porous structure. The conductivity, dielectric constant, hydrophilicity and swelling capacity of the PVA/PVP blends increased significantly with the addition of nHA. The higher the nHA content the higher the tensile strength and elongation of the composite nanofibers. Compared to the control blend, PVA/PVP, cell viability increased significantly by the presence of nHA, indicating an improved biocompatibility. It is clear from the data, that the addition of HA derivatives to polymeric matrices to play a major role in the mechanical performance of the electrospun nanofibers and cell response, firmly establishing these additives as essential for bone TE applications. Asran et al. proved just that by engineering a biodegradable nanocomposite scaffold made of PVA/Col and combined with 5 and 10 wt% unidirectional aligned nHA, to mimic the nanostructure of human bone tissue. Col is the main component of the bone organic matrix, being responsible for providing flexibility and resilience to the matrix. Addition of 5 wt% nHA resulted in scaffolds with uniform nanofibers, while scaffolds combined with 10 wt% exhibited agglomerates of nHA along the fibers’ axis. The fibers’ diameter suffered alterations as well, with those combined with 5 wt% nHA ranging between 100 and 530 nm and those with 10 wt% nHA ranging between 350 and 520 nm. Since the organic matrix fibers in the natural bone tissues have diameters varying from 100 to 450 nm, the addition of 5 wt% nHA was determined more efficient. Scaffolds containing 5 wt% exhibited also the highest tensile strength and elastic modulus, increasing the overall rigidity of the composite scaffold [109]. This study confirms the importance of HA-derivatives for bone composite production but demonstrates the need to control their ratio in the polymeric matrix. Their presence in the composite cannot be such to overcome the flexibility and resilience of the polymeric network, nor be insignificant to the mechanical performance and bioactivity of the overall scaffold.

The incorporation of magnetic nanoparticles and BG within electrospun scaffolds has been highly sought out for biomedical applications. Magnetite (Fe_3_O_4_) and maghemite (γ-Fe_2_O_3_), which have both high oxidative stabilities, have been shown to enhance the mechanical properties and the biocompatibility of PVA fibers. Cell proliferation rates have also been improved with PVA-γ-Fe_2_O_3_ combinations, revealing their promising biomedical potential as TE scaffolds [120,121]. BG-based electrospun nanomembranes prepared from combinations of PVA/BG 45S5 and PVA-magnetic bioglass (MBG) were established has highly bioactive bioactivity. Additionally, even though the resultant scaffolds were porous and possessed a textured surface, the BG and MBG additives increased the overall mechanical properties, including the ultimate tensile strength of the PVA nanofibers from 5.5 (PVA) to 20.9 MPa (PVA/BG) and 26.0 (PVA/MBG). Further, PVA/BG and PVA/MBG showed evidence of strong ion exchange with the pH of SBF reaching saturated values of 8.78, after 15 days of immersion. High pH values have been determined as necessary for new bone formation [122].

Core-shell electrospun nanofibers made of PCL and silk fibroin (SF) have been produced by co-axial electrospinning for a controlled and sustained release of growth factors derived from platelet-rich plasma (PRP). The shell of the fibers was produced from PCL/SF, while de core was prepared from PVA and calcium chloride (CaCl_2_)-activated PRP. SF was used as a “pore former” to enhance the controlled release of PRP and to reduce the viscosity of the shell material. Co-axial nanofibers containing 5 wt% PVA/PRP displayed uniform diameters and a metabolic activity superior to the control group. This increase was explained with the superior release of growth factors from the PRP, which promoted the hMSCs proliferation. In vivo testing demonstrated the PRP-loaded scaffolds to instigate new bone formation and the expression of Col II within bone defected sites, after 4 weeks’ post-surgery [123]. PRP has also been incorporated within PVA/poly(ether sulfone) (PES) composite scaffolds (PVA/PES/PRP) and its influence on the osteogenic differentiation of human adipose-derived mesenchymal stem cells was followed. Data showed that cells seeded onto the novel PRP-incorporated scaffold exhibited the highest alkaline phosphatase activity and calcium content. PRP enhanced cell proliferation and adhesion, and was proven effective in inducing osteogenesis due to a high concentration of different growth factors, including vascular endothelial growth factors, insulin-like growth factors, platelet-derived growth factors and transforming growth factor beta [124]. The addition of PRP to PVA-containing nanofibrous scaffolds is relatively recent. However, these studies show PRP in a very promising light particularly due to the elevated number of growth factors, associated with or instigated by it, which plays a major role in bone TE. In such cases, the polymeric matrices work only as a base substrate, with their physical and mechanical properties being the most relevant to the application.

### 4.2. Skin

The skin is the largest organ of the human body providing protection against dehydration, microbial invasion and external stimuli including mechanical, chemical, thermal and UV-light offenses [125].

Injuries to the skin are frequently caused by burns, trauma, chronic ulcerations and skin diseases, opening the biological environment to possible infections. Indeed, a moisture, warm and nutrient rich environment provides bacteria with optimal conditions to proliferate and colonize, ultimately resulting in infected wounds [126]. Skin grafting remains the conventional “gold standard” method to treat those severe wound conditions. However, a great number of limitations accompany this methodology, including donor site shortage, scarring at donor site, localized pain and elevated risk of rejection and infection. TE is a promising approach to restore the functions of wounded skin tissue [127] and to overcome the limitations associated with autografts, allografts and xenografts [128]. In recent years, nanofibrous scaffolds with a wide variety of physical and chemical properties have been selected for applications in wound healing, because of their high porosity and capacity to absorb exudates [126].

**Figure 6:**
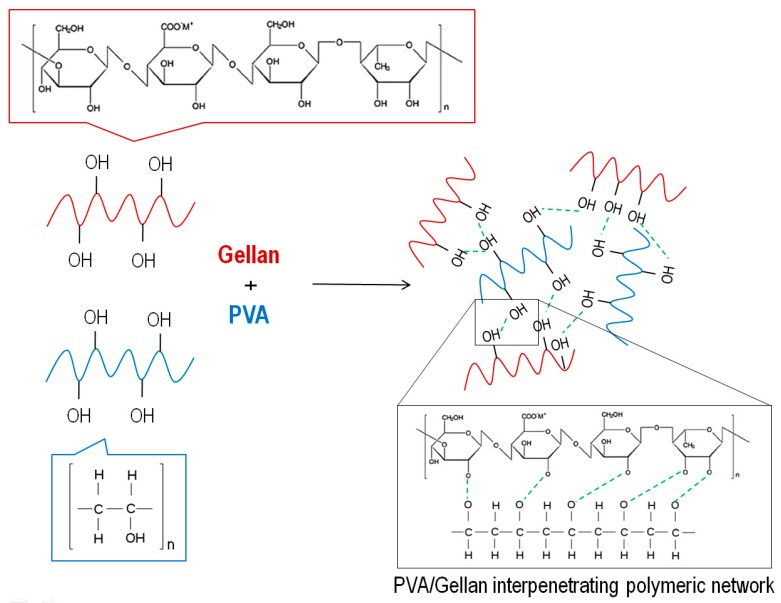
Representation of the interactions established between PVA and gellan, after crosslinking (adapted with permission from [129]).

For many years, PVA-based scaffolds have been applied for skin healing and reconstruction purposes. Vashisth et al. designed a hydrophilic scaffold based on the combination of PVA and gellan (PVA/gellan) for skin repair. Gellan, as a natural polymer, exhibits excellent properties to this purpose including biodegradability, biocompatibility and aqueous adsorption properties, making it a suitable candidate for TE. In the blend, PVA reduced the polymer repulsive forces, leading to the formation of uniform nanofibers. PVA/gellan nanofibers were physically crosslinked by heat treatment at 150 °C for 30 min. The interactions between the two polymers can be depicted in Figure 6. Cell culture studies using human dermal fibroblast (3T3 L1) determined these novel scaffolds as promoters of cell adhesion and proliferation compared to conventional gellan hydrogels and dry films [129].

**Figure 7:**
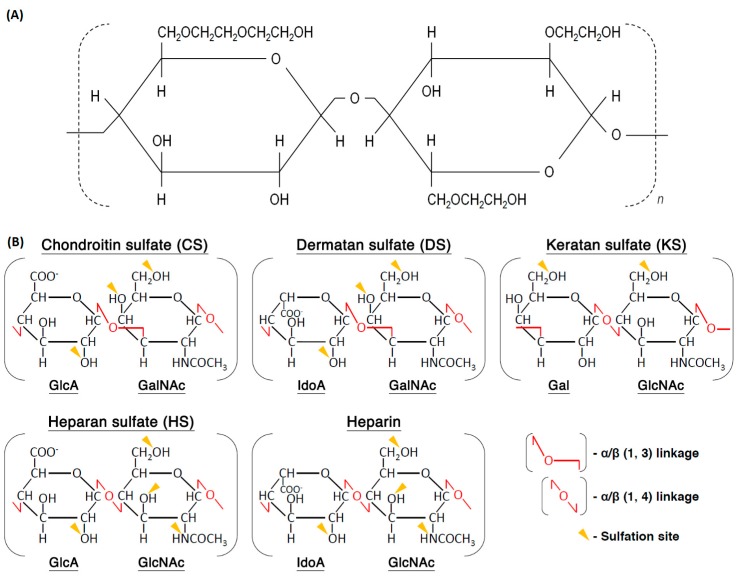
(**A**) Molecular structure of hydroxyethyl cellulose (HEC). (**B**) Structure of glycosaminoglycan chains (adapted with permission from [130]).

Scaffolds produced from PVA/HEC have also attracted much attention for skin applications, since the molecular structure of the HEC is very similar to that of the glycosaminoglycans (GAGs) present in the dermis (Figure 7). PVA facilitates fiber formation by reducing the HEC viscosity. The obtained constructs were crosslinked with GA. Zulkifli et al. observed that fibers containing more PVA exhibited a larger diameter and higher tensile stress and strain values, while those with an increased HEC content registered an improvement in their thermal stability. Overall, the nanofibrous scaffolds were uniform, porous and beadles [128]. Mechanical evaluations of the PVA/HEC scaffolds after 12 weeks of incubation in phosphate buffered saline solution (PBS) and Dulbecco’s modified Eagle’s medium (DMEM), showed that the constructs lost their structural integrity. Incorporation of Col within the scaffold reduced the degradation rate in both media. PVA/HEC/Col immersed in PBS exhibited the lowest modulus and tensile stress, most likely because of the high cleavage of polymer repeating units in the backbone linkages [97].

Gholipour-Kanani et al. developed a PVA/PCL/chitosan nanofibrous scaffold for applications in burns and excisional cuts. Scaffolds were tested with and without seeded hMSCs, which the goal was to recruit other host cells and induce the secretion of growth factors and matrix proteins. Scaffolds were implanted in vivo on the dorsum of rats, and the healing process was monitored. Cell-seeded scaffolds were found more successful in promoting wound healing than the acellular scaffolds. Here, the chitosan anti-inflammatory, antioxidant and antibacterial capacities reduced the number of inflammatory cells at the wounded site, and the physical and mechanical properties of the PVA and PCL maintained the integrity of the fibrous webs while in contact with blood and fibrin [40]. PVA and PCL have also been combined with gum tragacanth to fabricate 3D biodegradable nanofibrous scaffolds through two nozzles electrospinning. To this purpose, PVA/gum tragacanth blend solution was injected in one syringe and PCL in another. Once again, the presence of PVA and PCL in the formulation improved the spinning performance of the additive and the general mechanical properties of the fibrous construct. Hybrid nanofibers displayed greater strength and maximum strain than the individual polymers and PVA/gum tragacanth. The hydrophilic nature of the hybrid scaffolds promoted an effective fibroblast-like cells spreading and a 95.19% *Escherichia coli* (Gram-negative) reduction [126]. Combinations of PVA and chitosan are also very common in wound dressings. Adeli et al. developed a PVA/chitosan/starch nanofibrous scaffold, in which the carbohydrate biopolymer formed of amylose (20%–30%) and amylopectin (70%–80%) and endowed with biocompatible, biodegradable, nontoxic, high abundance and cost-effective characteristics, was used as additive. The hybrid formulation demonstrated great capacity to sustain a moist environment for wound regeneration, with balanced water absorption and water vapor transmission rates, and to protect the wounded area against external forces during the healing process. The scaffold excellent antibacterial activity against both Gram-negative, *Escherichia coli*, and Gram-positive, *Staphylococcus aureus*, bacteria was demonstrated. Moreover, in vitro cytotoxicity assays revealed appropriated cytocompatibility and cell viability, which was also corroborated via a scratch assay, confirming the remarkable potential of this polymer combination for wound healing [131]. Like starch, keratins, naturally abundant proteins found in animal tissues, including hair, wool, horns, hooves, nails, reptile scales and bird beaks and feathers, cannot be processed on its own via electrospinning; they require a base or matrix polymer to aid with the scaffold construction. Blends of PVA and keratin have demonstrated remarkable mechanical properties and biocompatibility. Increased keratin content in the blend has been shown to decrease the electrospun solution viscosity, leading to a reduction of the fiber diameter. PVA/keratin constructs are also known to promote cell adhesion and proliferation, with cells growing predominantly on the surface of the electrospun mats, with limited infiltration [132].

### 4.3. Cartilage

**Figure 8:**
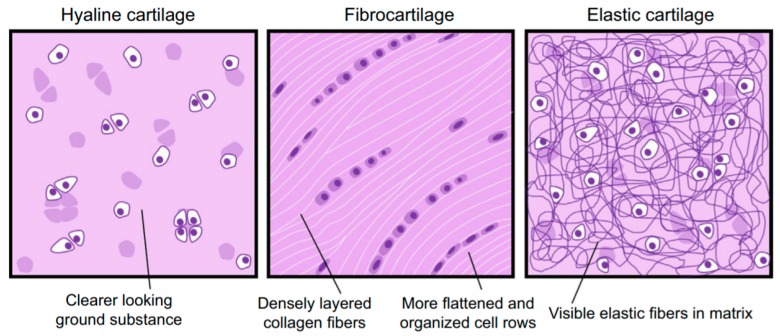
Histological differences between the types of cartilage: hyaline cartilage with rounded chondrocytes in lacunae; fibrous or fibrocartilage with thick, collagen bundles and rows of chondrocytes in between them and elastic cartilage with darkly stained elastic fibers visible in the matrix (adapted with permission from [136]).

Cartilage is a recognized vital tissue of the human body [133]. Cartilage is an avascular, aneural and alymphatic connective tissue that consists of chondrocytes and an extensive ECM, which is also produced and maintained by chondrocytes [134]. The ECM of cartilage is formed of a unique family of proteoglycans, enmeshed within a highly hydrated Col fibrillar network, and other several non-collagenous proteins, glycoproteins, lipids and phospholipids. Together, these components help retain water within the cartilage ECM, which is critical to maintain its unique mechanical properties [135]. Col is primarily responsible for the tensile strength, while the proteoglycans, entrapped within the different Col lattices, provide compressive strength [134]. The type of Col and proteoglycans, their organizational structure, abundance and distribution varies between the three types of cartilage: hyaline, fibrous and elastic; this conditions the cartilage appearance and biomechanical properties (Figure 8) [135,136]. Hyaline cartilage is the most common form of cartilage in the human body. It is found in the articulating surfaces of bones in synovial joints and in the ribs, nose, trachea, bronchi, larynx and growth plates [137]. Various Col molecules are expressed in the hyaline cartilage. Still, it is the Col II that accounts for 90%–95% of the total Col content, with the formed fibers being intertwined with proteoglycan aggregates [135]. Fibrous cartilage is primarily found in the intervertebral discs. Smaller amounts can also be found in the menisci, bone–tendon interfaces and ligament–tendon interfaces. It is mainly formed of Col I and of lower amounts of Col type VI and type II. The elastic cartilage is found in the external ears, larynx and epiglottis. The ECM of the elastic cartilage is predominantly made up of Col II, proteoglycans and elastin fibers. The elastin fibers are responsible for the yellowish appearance of the tissue and its increased elasticity [99].

Diseases affecting cartilage range from extremely common conditions such as osteoarthritis, which is estimated to affect as many as 37% of all adults in the USA [138], to rare genetic disorders such as spondyloepimetaphyseal dysplasia aggrecan type, which has only been reported in three patients worldwide [139]. The aging of the population, which causes the dissipation of chondrocytes from superficial regions, decreasing the hydration of the matrix and the size of proteoglycan aggregates within the ECM [135], trauma and accidents are some of the causes behind cartilage damage. The inherent ability to spontaneously repair cartilage is also limited to the low mitotic activity of its resident cells, the chondrocytes [136,140]. Surgical strategies to repair cartilage chondral or osteochondral defects have been developed to restore joint function and eliminate associated pain, including stimulation of the marrow by microfracture, mosaicplasty and cell-based therapies. Although these surgical strategies have demonstrated some degree of success, the regenerated tissue tends to be morphologically, biochemically and biomechanically inferior to the native cartilage [140]. Additional surgery is often required to regain complete function. There is an important need for new regenerative approaches that augment the success rates of the available repair processes and facilitate adequate tissue regeneration and longevity. Alternatives that resort to the combination of hMSCs seeded onto biomaterials loaded with appropriated growth factors have been suggested. In the last few years, a considerable array of materials and production methods have been explored to generate biomaterial scaffolds for cartilage TE [141].

Beadles, narrow sized photopolymerizable PVA scaffolds have been prepared by synthesizing methacrylated PVA (PVA-MA) in glycidyl methacrylate, followed by crosslinking with UV radiation in situ, and surface activation with 1,1-carbonyldiimidazole to promote Col immobilization. The scaffolds biocompatibility was examined against two cell lines, the 3T3 mouse fibroblasts and the human umbilical vein endothelial cells ECV304. Cell morphology observations, viability tests and monitoring of cells migration indicated that the Col-modified nanofibrous scaffolds were prone to cartilage regeneration. Even though both cell lines were compatible with Col-immobilized scaffolds, ECV304 cells showed higher viability [140,142,143]. Bi-layered composites mimicking the superficial and transitional zones of a cartilage tissue have also been produced using PVA and Col I. Random and aligned nanofibers of PVA/Col I blends were electrospun on top of a freeze-dried porous discs made of Col I and their physical, mechanical and biological properties were evaluated. The diameter of the aligned nanofibers was found significantly smaller and narrower than the random ones; however, their Young’s modulus and tensile strength were significantly superior. After primary chondrocytes culture, both composites’ Young’s modulus and ultimate tensile strength increased as well as the GAGs and Col II secretion. Generally, it was seen that the bi-layered aligned composite mimicked more closely the structure of a cartilage surface tissue than the randomly organized [144]. Due to the flexible and fibrous nature of cartilage and the high composition of Col in all different types, scaffolds made of PVA and Col blends are highly desirable in cartilage TE. As such, it is common to find many variations of the use of the two polymers in fibrous constructs. Mehrasa et al., for instance, reported the incorporation of inorganic nanoparticles made of zeolite and silica (nZe and nSi) within a matrix of PVA/Col. Zeolites, most commonly used as drug carriers or magnetic resonance imaging contrast agents, are biocompatible, capable of reducing TiO_2_-induced reactive oxygen species in fibroblast-like cell lines and of enhancing oxygen delivery to cells suffering hypoxia. On their turn, nSi incite biomineralization, while increasing the stiffness of polymers without decreasing their mechanical strength. Addition of nZe and nSi to PVA/Col scaffolds resulted in an improved Young’s modulus and tensile strength, increased porous size, and decreased in vitro degradation. There were no significant differences in the spreading of chondrocyte cells between nZe- and nSi-modified scaffolds, with both contributing to an improved cell adhesion [145].

PVA/chitosan scaffolds reinforced with calcium carbonate (CaCO_3_) nanoparticles (from 1 to 5 wt%) were engineered for an enhanced mechanical performance and biocompatibility. Chitosan has been shown to stimulate hyaluronan by providing a suitable environment for chondrocytes propagation, while CaCO_3_ aside from being non-cytotoxic and conferring great mechanical strength is known to facilitate cell proliferation. ATDC5 mouse chondrogenic cell line (derived from teratocarcinoma AT805), which is an excellent model of cartilage formation for osteoarthritis, was cultured onto three different networks of nanofibers, PVA/chitosan, PVA/chitosan/CaCO_3_ and PVA/chitosan/HA fibers. All were seen to support ATDC5 cells growth even though, by increasing the roughness of the matrix, PVA/chitosan/CaCO_3_ (4 wt%) provided the most suitable environment [146]. Hybrid nanofibers of PVA/chitosan/gelatin (GN) were found more suitable for KP-hMSCs (an immortalized hMSCs line) attachment and proliferation because of their similarity to the ECM, namely high surface area and porosity, that stimulated transport of nutrients and metabolites [147]. Using a PVA/GN base matrix, Sadeghi et al. incorporated chondroitin sulfate, the main sulfated GAGs present in the ECM of soft connective tissues, and followed the L929 fibroblasts-like cells viability. Data revealed a suitable interaction with the crosslinked PVA/GN/chondroitin (15 wt%) nanofibers, with an excellent proliferation rate without any indication of cytotoxicity [148]. These investigations established the potential of electrospun PVA/chitosan and PVA/GN hybrids for cartilage restructuration because of their enhanced strength, stability and cytocompatibility.

Even though combinations like PVA/Col or PVA/chitosan are expected in cartilage TE, there are others less explored and more unexpected. For instance, PVA/biodegradable waterborne polyurethanes (BWPU) were successfully prepared at different ratios, free of organic solvents, by Wu et al. PU has been broadly used in the biomedical field because of its excellent biocompatibility, appropriate microstructure and exceptional mechanical properties. However, its synthesis requires organic solvents, which may compromise in vitro cell culture or in vivo implantation. As such, waterborne BWPUs, with suitable mechanical properties, adjustable degradation rates, hemocompatibility and cytocompatibility, were prepared engineered and their interaction with PVA examined. As the content of BWPU increased in the blend, the average fiber diameter and tensile modulus decreased; however, seeding and growth of L929 fibroblast-like was more efficient [149]. Another less explored blend is PVA with SF. Silk is a biodegradable, widely researched, FDA (Food and Drug Administration)-approved material for TE applications. SF has an edge over other natural fibers as a scaffolding material due to its high mechanical strength, flexibility, biocompatibility and controlled biodegradability, which is suitable for load bearing. SF has a good compatibility with living tissues and good oxygen permeability that can enhance cell attachment and proliferation. Pillai et al. developed PVA/SF nanofibrous scaffolds and investigated the attachment and proliferation of primary human meniscal cells. Data revealed higher cell attachment, proliferation of meniscal cells, aside from superior DNA and Col contents compared to PVA or SF individual scaffolds [150]. These new or less explored combination of materials are of extreme importance to the development of improved strategies for cartilage regeneration in the future.

### 4.4. Vascular Grafts

Cardiovascular diseases are a leading cause of death worldwide, in particular the coronary artery disease, which affects small and medium-sized blood vessels and accounts for 53% of the total mortality rates [151].

Vascular transplantation is an effective clinical strategy. However, its application is often limited by donors shortage [152]. Currently, the available alternatives to treat these conditions resort to autologous grafts (e.g., coronary artery bypass graft with autologous mammary arteries and saphenous veins), allografts (donor/cadaveric), xenografts (e.g., bovine or porcine pulmonary valve conduit), artificial prostheses or synthetic vascular grafts made of expanded polytetrafluroethylene (ePTFE) and polyethylene terephthalate (PET) [96]. Still, the use of autografts and allografts is limited due to the lack of tissue donors, previous harvesting or anatomic variability. Xenografts have also a relatively short life span. As an example, a bovine or porcine graft may resist for up to 15 years, which for pediatric patients means a new implant replacement and new surgical risks in 10–15 years time. Other issues include poor control over the implant physical and mechanical properties, inflammation and calcification. Artificial grafts are highly demanded [153], however they may also raise some serious concerns including increased risk of infection, thrombogenicity, lack of growth potential and possibility of rejection by the immune system within a few months, if the diameter of the vessel is smaller than 6 mm [154]. Hence, there is an urgent need for alternative approaches based on functional small-diameter vascular grafts for clinical arterial replacement.

To respond to this acute clinical demand, Parikh et al. designed and fabricated a novel small diameter helical vascular scaffold that raised the survival of endothelial cells (ECs) above the conventional tubular scaffold. Parikh et al. designed an intelligent system that replicated the natural structure and hemodynamics of small arteries. Using computational fluid dynamic simulation and the electrospinning technique, a new scaffold structure with a small diameter of 6 mm was designed with inner helices, to enable a ‘spiral flow’ effect with an improved shear stress profile. They recreated the natural environmental conditions to ensure ECs survival and growth, and consequently increased long-term graft patency. These nanofibrous tubular scaffolds were produced from combinations of PVA and GN, which enhanced cell viability and displayed an improved responsiveness to shear stress while retaining the physiological levels of intravascular pressure. Hence, in addition to showing potential as grafts for preclinical testing, these helical scaffolds may also serve as a tool to study ECs behavior under an environment that mimics closely the natural structure and behavior of real vessels [151]. The incorporation of PCL within PVA/GN matrices and the functionalization with heparin (reduced the risk of thrombosis) resulted in a scaffold with large fiber diameters, improved tensile strength, Young’s modulus and elongation results well above the natural porcine coronary artery. The heparin immobilized improved the constructs’ anticoagulant features and the ECs growth. Heparin is known to prompt cells to secrete the vascular endothelial growth factor, which stabilizing effect is beneficial for cell growth. The presence of PVA in the blend was responsible for increasing the scaffolds porosity, which allowed more space and freedom for cells to attach, arrange their structure and ultimately grow, thus explaining the higher cell proliferation registered on PVA/PCL/GN compared to PCL/GN fibers [153].

PVA/PET composites have also been tested for applications in vascular grafts. PET is a semi-crystalline polymer, which electrospun nanofibers are useful for protective clothing, filtration devices, tissue scaffolding and, most importantly, to produce membranes for vascular grafts. Different ratios of PVA/PET were tested. Here, PVA worked as an additive to increase the viscosity and spinnability of the polymeric blend and to improve fiber morphology. Examinations of water flux-pressure revealed the superhydrophilic properties of the composite scaffold. Mechanical properties were also improved by the addition of PVA, resembling those necessary for vascular graft implants [155]. These results suggest that superhydrophilic fibrous constructs, highly desirable in vascular TE, may be easily fabricated from combinations of hydrophobic polymers and smaller amounts of hydrophilic. Even though in bone, skin and cartilage TE most fibrous scaffolds used PVA as matrix polymer or base substrate, in vascular TE only small amounts are required. PVA causes a great impact in the final construct but until a certain amount; after that point, its presence becomes inefficient or even prejudicial, conditioning for instance the mechanical stability of the final scaffold.

### 4.5. Nervous Tissue

Nerve diseases namely acute injuries, such as peripheral nerve injury, spinal cord injury and traumatic brain injury, and chronic injuries like neuro-degeneration diseases, can cause various function disorders of the nervous system related to memory and voluntary movement [156].

The central nervous systems (CNS) functions include carrier and interpreter of signals, as well as a generator of excitatory stimuli to the peripheral nervous system (PNS). The five main components of the CNS are brain, spinal cord, optic, olfactory and auditory systems. The PNS, formed of a collection of nerves, sensory receptors and ganglia, is one of the largest and most complex structures in the body [157]. Neurons and neuroglia are two of the main cell categories in the nervous system. Neuroglia are support cells, which include Schwann cells in PNS, and astrocytes and oligodendrocytes in CNS. Neuroglia are more abundant than neurons and have some capacity for cell division. Although neurons cannot divide by mitosis, they can partially regenerate under certain conditions. Neurons have a very limited capability of regeneration [158]. However, under the right conditions, axon extensions can regenerate over small gaps caused by injury, reconnecting with the distal stump and eventually re-establishing their functions. In the case of small injuries, current treatments for severed nerves typically rely on micro suture of the nerve stumps [159]. Yet, if substantial loss of nervous tissue occurs, clinical treatment requires nerve transplantation from a second operative site in the patient, such as an autologous nerve graft, vein graft or arterial graft [160]. This method is very far from being the “gold standard”; its benefits have to be counterbalanced with the loss of functions at the donor sites, potential infections, possible formation of painful neuromas, structural differences between donor and recipient grafts hindering regeneration and shortage of graft material for extensive repair [159]. Nutrients supply to the grafted nerve also needs to be considered since large defects require donor nerves with an intact vascular input that can be connected to the existing blood supply at or close to the lesion site [160].

Production of implantable scaffolds capable of bridging long gaps with results similar to autografts, without requiring the harvest of autologous donor tissue, is a major challenge in nerve TE. It generally involves the seeding of cells and bioactive molecules such as growth factors onto a 3D biodegradable and biocompatible scaffold, which is grown in vitro prior to implantation [161]. To simulate the native nerve tissue, scaffolds are required to display appropriated mechanical characteristics while providing enough flexibility. Furthermore, artificial nerve grafts need to present electrical conductivity to accelerate axonal elongation on the charged surface and promote transfer of electrical stimuli for an improved regeneration [18]. 3D microstructured scaffolds are desired for neural tissue regeneration [22] since most neural cells are in the micron scale, thus requiring larger pore size systems to allow cell infiltration [161].

**Figure 9:**
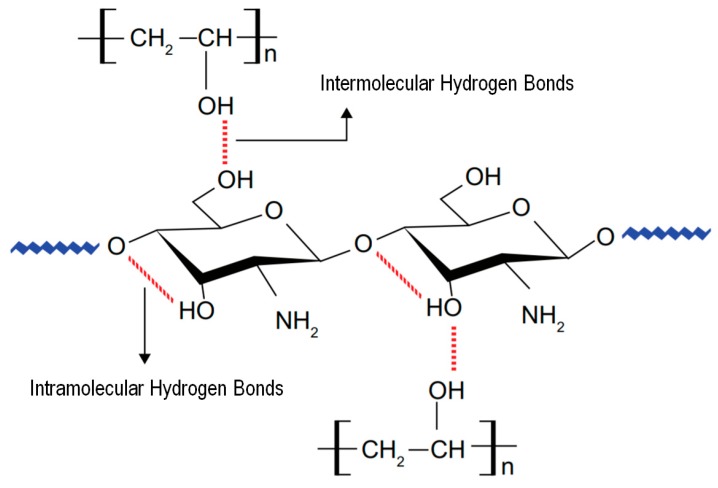
Schematic representation of the intermolecular and intramolecular hydrogen bonds generated with blending of PVA and chitosan (adapted with permission from [22]).

In an attempt to find new therapies, Alhosseini et al. designed a PVA/chitosan hybrid construct with numerous and large pores (Figure 9). Chitosan reduced the swelling of the scaffold, from 450% (bare PVA) to 300% (hybrid), and the degradation rate. This happens, because its amine groups are more reactive towards GA during crosslinking than towards the PVA hydroxyls, thus increasing the density of the crosslinking and, consequently, slowing down depolymerization. In vitro testing, demonstrated the PVA/chitosan scaffolds to instigate PC12 nerve cells adhesion, proliferation and migration [22]. Nerve growth factor (NGF) has been incorporated at different concentrations (5, 10 and 20 wt%) onto PVA/chitosan nanofibers to mimic the biochemical properties of the neural tissue. NGF has been shown to enhance peripheral nerve regeneration and protect neurons from injury-induced death. A uniform and continuous web of nanofibers, required for a homogenous cell growth, was produced. Human neuroblastoma SKNMC and human glioblastoma-astrocytoma U373 cells adhered and proliferated preferentially on the PVA/chitosan scaffolds conjugated with NGF in comparison with the controls (positive—nanocomposites without NGF and negative—polystyrene). Among the tested formulations, the nanocomposites endowed with 5 and 10 wt% of NGF promoted the most proliferation and physical attachment [161]. Instead of NGF, Shokrgozar et al. proposed the incorporation of single-walled carbon nanotubes (SWCNT), at different concentration, on electrospun PVA/chitosan nanofibers to improve the cell response of neural tissues. Carbon nanotubes (CNTs) have been used in neural TE due to their high conductivity and mechanical strength, which improves neural signal transfer and cell adhesion. The polymeric constructs containing 17% SWCNT, showed improved mechanical performance despite the elevated porosity (73%). In terms of biocompatibility, no differences were detected between PVA/chitosan/SWCNT nanocomposites and the control groups, namely the thermoplastic starch (negative control) and PVA/chitosan (positive control). Generally, the proliferation rate of brain-derived cells and U373 cell line in nanocomposite extraction solutions was similar to the control groups. However, data showed a clear cell preference for the PVA/chitosan/SWCNT, with an improved cell viability after 7 days of culture [162]. Experiments have also been performed to study the incorporation of multi-walled carbon nanotubes (MWCNT) on PVA/chitosan nanofibers. Smooth and uniform PVA/chitosan and PVA/chitosan/MWCNTs nanofibers, stable in water electrospun and crosslinked with GA vapor, were prepared. The MWCNTs increased the diameter and density of the nanofibers together with the tensile strength and Young’s modulus, while decreasing the scaffold’s porosity and brittleness. Protein adsorption was significantly higher on the PVA/chitosan/MWCNTs for the totality of the testing periods. In vitro cell culture of mouse fibroblasts L929, present in the perineurium (a layer of axons) and the Col, was significantly improved with the incorporation of MWCNTs. Another study combined BG nanoparticles with PVA/chitosan/CNTs nanocomposites. The presence of BG nanoparticles within the polymeric structure increased its biological activity while altering its mechanical properties. Data suggested that the presence of BG particles to decrease the overall flexibility of the scaffold even though the tensile strength increased. Scaffolds containing BG nanoparticles were effective in promoting proliferation and bioactivity of P19 embryonic carcinoma stem cells [163]. From the literature, it is clear the electrospun scaffolds made of PVA and chitosan blends to offer the most advantages for nerve TE. Still, additives are effective in providing specific cell responses that contribute actively for successful tissue regeneration.

In alternative to PVA/chitosan, Golafshan et al. engineered a hybrid scaffold of PVA/alginate incorporated with graphene (Gr) to mimic the ECM of the peripheral nerve. Alginate is a non-toxic, biodegradable and biocompatible polymer that combined with the remarkable physicochemical properties of Gr generates systems desirable for nerve TE applications. Incorporation of Gr nanosheets within the polymeric blend did not alter the fibers morphology but increased the hydrophobicity of the scaffold, consequently delaying the degradation rate. It was noted that after physical and chemical crosslinking the average fiber diameter increased and the scaffolds, particularly those treated with 1 wt% Gr, displayed superior mechanical properties including tensile strength, toughness and elongation. In vitro testing, demonstrated the PVA/alginate/Gr matrices to effectively improve the initial attachment, spreading and proliferation of PC12 nerve cells and that to be intimately related to the scaffolds intrinsic electrical and mechanical properties [18].

### 4.6. Corneal Tissue

Cornea is a transparent, avascular and multilaminar structure of the ocular surface that forms a barrier to protect the intraocular structure from the environment, while refracting light onto the retina. The cornea is divided in five distinct layers, the corneal epithelium (outermost layer), Bowman’s layer, the stroma, Descement’s membrane and the corneal endothelium (innermost layer) [164]. Keratoconus, bullous keratopathy and scarring are some of the injuries or diseases that affect the cornea region and may eventually cause visual impairment or even blindness. Globally, there are over 10 million people suffering from corneal blindness (the second leading cause of loss of vision after cataracts). Still, only 120,000 corneal transplants (in average) are undertaken annually [103]. The cornea transplantation is considered the only effective treatment for irreversible corneal damage [165]. However, the supply of donor corneal tissue worldwide falls well short of the demand, and when available corneal grafts may trigger the host immune response, resulting in tissue rejection, or even transfer diseases from unhealthy donors. These complications are compounded by the growing use of corrective eye surgery, which renders these corneas unsuitable for grafting, further reducing the availability of acceptable allogenic supplies [166]. To overcome these problems, researchers have attempted to fabricate scaffolds with similar strength and transparency of the native cornea for corneal replacement [167]. Successful corneal TE requires appropriate scaffolds where the cells can proliferate, organize their ECM, and replicate the corneal native structure and functions [166]. In addition to biocompatibility, electrospun scaffolds for corneal TE are required to degrade at a rate similar to the regeneration rate of the native ECM, possess mechanical properties that match those of the human cornea (elongation at break and tensile strength up to 0.19 and 3–5 MPa, respectively), improved scaffold transparency and low opacity to guarantee the visual functions [103].

As a synthetic polymer, PVA has been widely used as a supporting material for corneal TE due to its transparency, flexibility and mechanical stability [23,94]. Natural polymers are not as frequent since this combination of properties is difficult to attain. Still, Col remains a very important additive, widely sought out for corneal scaffolding production, since aside from its good biodegradability, excellent biocompatibility and low immunogenicity, it is also the main component of the natural corneal ECM [168].

**Figure 10:**
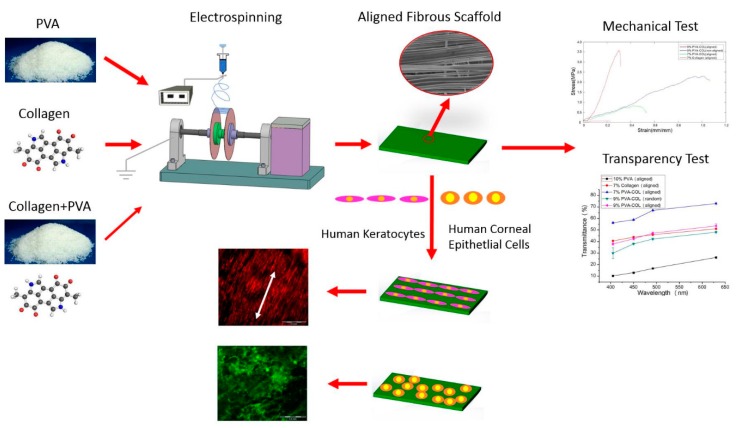
Representation of the production and characterization process of PVA/Col scaffolds for corneal applications (adapted with permission from [23]).

Random and aligned PVA/Col electrospun scaffolds were inoculated with human keratocytes (HKs) and human corneal epithelial cells (HCECs) and cultured up to four weeks (Figure 10). Aligned composites displayed smaller, uniform fibers compared to the random, and a tensile strength of 3.581 MPa, which was approximated to the natural corneal tissue (3–5 MPa). Light transmittance also increased with the alignment of the nanofibers. Both HKs and HCECs adhered and proliferated well on the PVA/Col scaffolds. In fact, the aligned nanofibers induced an orderly HK growth, mimicking more closely the corneal tissue [23]. PVA has been studied to improve the durability of the corneal epithelium layer. To accomplish that, PVA has been activated with isocyanate groups from hexamethylene diisocyanate (HMDI) and functionalized with Col I by immersion. After swelling, the PVA/Col scaffolds became translucent. Attachment of corneal epithelial cells and corneal stromal cells was enhanced by this polymeric blend. Corneal epithelium cells were well differentiated, and the stability of the corneal epithelium layer improved significantly. However, further work is required since the engineered scaffold did not achieve satisfactory levels of light transmittance [169]. In fact, on of the main issues with corneal scaffolding production is the capacity to maintain polymer light transmittance after processing. Seyed et al. tried to overcome this issue by replacing Col with chitosan. They prepared PVA/chitosan blended scaffolds crosslinked with 1-ethyl-3-(3-dimethyl aminopropyl)-carbodiimide (EDC) and 2 N-hydroxysuccinimide (NHS) to serve as in vitro carriers for human limbal stem cells delivery. These cells are responsible for maintaining and regenerating the corneal epithelium throughout life and may also act as a “barrier” to conjunctival epithelial cells by preventing their migration to the corneal surface. Optical clarity tests were conducted revealing PVA/chitosan with an 88% optical transparency against the 72%–82% of standard cornea (positive control) or the 78% of the human amniotic membrane. PVA/chitosan scaffolds degraded at a much slower rate than pristine PVA and increased the swelling volume three times over. By increasing the amount of chitosan in the blend, the strength of the scaffold also increased. In vitro testing demonstrated a good cell proliferation and growth on the hybrid scaffolds [170].

### 4.7. Other Applications

PVA-based scaffolds represent a niche when it comes to kidney regeneration and insulin production. Yet, has additive or stabilizing agent, PVA has been known to promote cell differentiation while stimulating regeneration.

Poly lactic acid (PLA) has enjoyed great importance as a bioactive candidate for TE. However, its hydrophobic nature and its weak mechanical properties, with poor ductility and low strength, have hindered its practical use. Alharbiet al., resorting to electrospinning, fabricated four different scaffolds: (1) core-shell structure with PVA in the core and PLA in the shell, (2) core-shell structure with PLA in the core and PVA in the shell, (3) pristine PLA and (4) pristine PVA, all for prospective applications in kidney TE. Between the four, the core-shell composite with PLA in the core and PVA in the shell was considered the most stable structurally, mechanically, with great wettability and biocompatibility properties. In the opposite formation (PVA core and PLA shell), the brittle nature of PLA led to the appearance of broken areas within the shell. PLA/PVA core-shell composite scaffolds increased cell attachment and improved cell growth of human embryonic kidney cells HEK-293. The new co-axial PLA/PVA scaffolds revealed great potential as candidates for biomedical applications, particularly for kidney regeneration [171].

Combinations of these synthetic scaffolds also enjoy a special reputation in restoring and maintaining damaged pancreas functions. A study was designed to evaluate the differentiation of human induced pluripotent stem cells (hiPSCs) into insulin producing cells (IPCs) using PVA and poly-L-lactic acid (PVA/PLLA) nanofibrous scaffolds. HiPSCs are an appropriate cell source to study regenerative therapies because of their high potential of self-renewability and differentiation as well as low immunogenicity. Enderami et al. optimized the PVA/PLLA construct surface hydrophilicity by applying oxygen-induced plasma treatment. Data demonstrated that this scaffold could support homogenous and efficient differentiation of hiPSCs into IPCs [172]. Differentiation of iPSCs was also evaluated on PVA/PCL scaffolds, by means of stimulation of glucose-responsive beta-like cells. Here, a significant increase in the secretion of insulin upon glucose stimulation on PVA/PCL scaffolds was verified. These findings show that mature IPCs can be generated from iPSCs in 3D cultures. Besides, differentiation on these 3D systems was proven more efficient than monolayer cultures, which was confirmed by the increasing levels of messenger RNA (mRNA), protein, insulin and C-peptide secretions. These nanofibrous scaffolds can be considered as feasible alternatives to the native ECM and become very useful for pancreatic TE. Still, more research is required to investigate the exact cellular and molecular mechanisms that take place at the interface of these scaffolds in vivo [16].

## 5. Conclusions

TE is one of the most exciting interdisciplinary and multidisciplinary research areas and is growing exponentially with time. Electrospun scaffolds are crucial in TE. Fibrous scaffolds can present aligned ultrafine fibers capable of encapsulating functional biomolecules that play an important role in tissue regeneration. Electrospinning allows the control of the morphology, porosity and fiber diameter of the scaffold by adjusting the processing parameters using simple and low maintenance settings. These characteristics make the electrospinning technique particularly suitable for the production of biomimetic nanofibrous scaffolds, mimicking the features of the native ECM, and providing a promising strategy to restore, maintain or improve tissue functions in different body regions: bone, skin, cartilage, vascular, neural, corneal and others.

PVA has been used as a base material or backbone and has been combined with other natural and synthetic origin materials to produce electrospun scaffolds with improved wettability, mechanical performance, thermal stability and biocompatibility. Through a careful and in-depth analysis, the role of PVA as a suitable candidate for TE was established. Processing and optimization steps for a successful polymeric scaffold production were reviewed. Literature shows that PVA nanocomposites are widely explored in TE and will continue being investigated in the foreseeable future. However, there are still fundamental questions about the behavior of PVA in blends that should be answer in the future to guarantee the TE 3D scaffolds diversity of multifunctionalities and to control the structural complexity and interfacial interactions for faster tissue regeneration. These issues require refinement of the electrospinning experimental parameters, boosting of systematic approaches, and advancement of specific knowledge. Future research should also focus on the application of PVA-based constructs in vivo, to truly comprehend this polymer behavior in living systems. Further, considering the relative low impact this polymer and technology have in the environment, it would be extremely important to invest in the optimization of its processing conditions and effectiveness for large scale production in detriment of other conventional, non-green approaches.

## Figures and Tables

**Table 2:** Effect of the molecular weight and degree of hydrolysis on the properties of PVA [87].

	Increasing	Decreasing
**Molecular Weight**	Viscosity	Flexibility
	Tensile Strength	Water Sensitivity
	Water Resistance	Ease of Solvation
	Solvent Resistance	
	Dispersing Power	
	Adhesive Strength	
	Block Resistance	
**Percentage of Hydrolysis**	Tensile Strength	Flexibility
	Block Resistance	Dispersing Power
	Water Resistance	Water Sensitivity
	Solvent Resistance	Adhesion to Hydrophobic Surfaces
	Adhesion to Hydrophilic Surfaces	

**Table 3:** Classification of PVA crosslinkers and respective applications [92].

Crosslinker	Structure	Functionality	Applications
**Glutaraldehyde**	Aliphatic dialdehyde	Two–CHO groups	Reducing oxygen permeability.
			Drug delivery applications
			Proton exchange membrane
			Ultrafiltration membranes
			Pervaporation systems
			Coating
**Maleic acid**	Aliphatic dicarboxylic acids	Two–COOH groups	Separation processes
**Fumaric acid**			Pervaporation systems
**Malic acid**			Pervaporation systems
**Sulfosuccinic acid**			Proton and methanol transport
**Phthalic acid**	Aromatic dicarboxylic acids		Development of polysulfone (PS) membranes
**Iso-phthalic acid**			
**Terephthalic acid**			
**Aconitic acid** **(cis and trans)**	Aliphatic tricarboxylic acids	Three–COOH groups	Development of polysulfone membranes
**Citric acid**			Support membrane for polysulfone
**Hexamethylene diisocyanate**	Aliphatic diisocyanate	Two–NCO groups	Improved thermal and mechanical properties of PVA
**Boric acid**	Non-linear	Three–OH groups	Improved melting behavior of PVA
